# CircRhoC promotes tumorigenicity and progression in ovarian cancer by functioning as a miR‐302e sponge to positively regulate VEGFA

**DOI:** 10.1111/jcmm.14736

**Published:** 2019-10-22

**Authors:** Li‐Li Wang, Zhi‐Hong Zong, Yao Liu, Xue Guan, Shuo Chen, Yang Zhao

**Affiliations:** ^1^ Department of Gynecologic Oncology Research Office The Third Affiliated Hospital of Guangzhou Medical University Guangzhou China; ^2^ Department of Gynecology The Third Affiliated Hospital of Guangzhou Medical University Guangzhou China; ^3^ Key Laboratory for Major Obstetric Diseases of Guangdong Province Key Laboratory of Reproduction and Genetics of Guangdong Higher Education Institute in Guangdong Province Guangzhou China; ^4^ Department of Gynecology The First Affiliated Hospital of China Medical University Shenyang China

**Keywords:** circRhoC, miR‐302e, ovarian cancer, VEGFA

## Abstract

Ovarian cancer is a leading cause of deaths due to gynaecological malignancy. While endogenous non‐coding circular RNAs (circRNAs) in cancer have attracted attention, their roles in ovarian cancer are not known. We used qRT‐PCR to quantify expression of circRhoC in ovarian cancer tissues and normal tissues. The effects of overexpressing or destruction of circRhoC on the phenotype of ovarian cancer cells were assessed both in vitro and in vivo. Dual‐luciferase reporter assay assesses the microRNA sponge function of circRhoC. Western blotting was used to confirm the effects of circRhoC and microRNA on target gene expression. Our results showed that circRhoC was significantly up‐regulated in ovarian cancer tissues compared to normal ovarian tissues. Overexpression of circRhoC in CAOV3 ovarian cancer cell increased cell viability, migration and invasion ability; destroying circRhoC in A2780 had the opposite effects and inhibited ovarian tumour cell A2780 dissemination in the peritoneum in vivo. We confirmed circRhoC functions as a sponge for miR‐302e to positively regulate *VEGFA*; FISH experiments showed that circRhoC could co‐focal with miR‐302e; besides, overexpression of miR‐302e reversed the ability of circRhoC to positively regulate VEGFA, and what's more, RIP assay showed that circRhoC could directly bind with VEGFA; besides, VEGFA expression level in ovarian cancer tissues was positively associated with circRhoC expression. In conclusion, the oncogenic effect of RhoC in ovarian cancer is at least in part due to circRhoC, which functions not only as a miR‐302e sponge to positively regulate VEGFA protein expression, but may also directly bind and modulate VEGFA expression.

## 1 BACKGROUND

Epithelial ovarian cancer (EOC) is one of the most common gynaecological malignancies. Most patients are diagnosed with advanced stage disease, as EOC is frequently asymptomatic. Unfortunately, up to 60%‐70% of patients with advanced EOC suffer recurrence—even after positive surgical resection combined with chemotherapy—and have a poor prognosis.^1^ Therefore, detailed investigations of the molecular mechanisms of ovarian cancer tumorigenesis and progression are required to enable early diagnosis, provide targeted therapy and improve patient survival and quality of life.

Non‐coding RNAs, including microRNAs (miRNAs), long non‐coding RNAs (lncRNAs) and circular RNAs (circRNAs), do not encode proteins, but play crucial roles in epigenetic regulation and affect multiple biological processes.^2^, ^3^ MiRNAs, a class of small endogenous non‐coding RNAs that bind to the 3′ untranslated region (UTR) of target mRNAs to mediate mRNA cleavage or inhibit translation, have been shown to participate in numerous biological functions.^4^ The mechanisms that regulate miRNAs are complex and diverse, and the circRNA‐miRNA regulatory network is a hot spot of research.^5^

A recent study showed that precursor mRNAs transcribed from DNA can form covalently closed circular RNA molecules by back‐splicing of exons.^6^, ^7^ During back‐splicing, ALU repeats (ie the AGCT recognition sequence of the restriction endonuclease Alu I) within the introns upstream and downstream of exons are cyclized by complementary base pairing, and the introns are excised to form a circRNA^8^ Closed circRNAs frequently contain miRNA binding sites and can function as sponges to sequester miRNAs and inhibit their function.^9^ Those circRNAs composed of exons are more stable and have stronger adsorption capacity for miRNAs than linear mRNAs or lncRNAs. Therefore, circRNAs can act as efficient competitive endogenous RNAs to alleviate miRNA‐mediated inhibition of target genes.^10^

Several studies have shown circRNAs participate in the development and progression of cancer by acting as miRNA sponges.^11^ The oncogenic or tumour‐suppressive characteristics of circRNAs may be the same as those of the parent gene, or not. For example, circRNA_100269 and the mRNA of its linear isomer latrophilin 2 (*LPHN2*) are both down‐regulated in gastric cancer.^12^ CircBANP is overexpressed in colorectal cancer, while BTG3‐associated nuclear protein (*BANP*; also known as SMAR1) inhibits cyclin D1 gene suppression and functions as a tumour suppressor.^13^ However, knowledge of the expression and function of circRNAs in ovarian cancer is limited.

Ras homolog gene family member C (RhoC), a member of the Ras superfamily of oncogenes,^14^ is up‐regulated in a variety of cancers.^15^, ^16^ Overexpression of RhoC promotes progression and is associated with poorer survival in pancreatic ductal adenocarcinoma.^17^, ^18^ RhoC has also been confirmed to function as a key molecule required for invasion and metastasis in head and neck squamous cell carcinoma and gastric cancer.^19^, ^20^ We discovered RhoC is an oncogene that promotes tumorigenesis in ovarian cancer.^21^ Mechanistically, RhoC combines with and activates the effector ROCK to stimulate cell mobility, but also influences the progression of ovarian cancer by up‐regulating vascular endothelial growth factor A (VEGFA) and other protein expression.^22^, ^23^

We were curious to investigate if the *RhoC* transcript could form a circular structure by back‐splicing. Bioinformatic analysis of the *RhoC* mRNA using http://circbase.org/ revealed *RhoC* could potentially form 14 circular RNAs. We designed 11 primer pairs to identity the corresponding back‐spliced sites, and found circ_0013549 (hereafter referred to as circRhoC) formed from exons 4‐6 was expressed in ovarian cancer tissues and cells, and the sequence information was showed in the supplementary table. The purpose of this study was to explore the expression, function and potential mechanism of action of circRhoC in ovarian cancer.

## 2 MATERIALS AND METHODS

### 2.1 Ovarian cancer tissues and cell lines

Human ovarian cancer tissues (n = 127) and normal ovarian tissues (n = 24) were collected from patients who had undergone surgical resection at the Department of Gynecology, the First Affiliated Hospital of China Medical University (Shenyang, China). Two pathologists pathologically confirmed all of the specimens independently. Official approval for this study was obtained from China Medical University Ethics Committee (No: 2014‐27), and the study was conducted following ethical and legal standards. Ovarian cancer cell lines were purchased from the ATCC.

### 2.2 Culture and transfection of ovarian cancer cells

A2780 cells were cultured in Dulbecco's modified Eagle's medium (DMEM) supplemented with 1% penicillin/streptomycin and 10% foetal bovine serum (FBS) at 37°C in a 5% CO_2_ atmosphere, and CAOV3 cells were cultured in RPMI‐1640 (HyClone) at the same conditions_._ Cells were transfected with Lipofectamine 2000 (Invitrogen) and selected using puromycin. The sequences of the circRhoC‐overexpressing plasmid are provided in Table S3.

### 2.3 Cell viability assay

100 μL of medium contain 3000 cells were added into 96‐well plates, cultured to adherent. At 0, 24, 48 or 72 hours, 20 μL of 5 mg/mL tetrazolium (MTT; Solarbio) was added, incubated for 3 hours, dimethyl sulphoxide (DMSO) was added to dissolve the formazan precipitates, and the OD values used to calculate the cell viability were determined using a microplate spectrophotometer (BioTek Instruments).

### 2.4 Cell migration assays

Scratch wounds were created in 80% confluent cell monolayers using 200‐µL pipette tip, and then, the monolayers were cultured in FBS‐free media containing 20 µg/mL mitomycin. At 0, 24 and 48 hours, the wounds were photographed and measured using ImageJ software (National Institutes of Health). Cell migration was determined as (area of original wound − area of wound at different times)/area of original wound × 100%.

### 2.5 Cell invasion assay

Transwell chamber filters (BD Biosciences) were coated with 30 µL of Matrigel basement membrane (1:10); then, 200 µL of FBS‐free media containing 4 × 10^4^ cells was added in the top chamber and 600 µL complete media was added to the lower compartment. After 48 hours, the cells invaded to the back of the upper apartment were stained using crystal violet and counted under a light microscope.

### 2.6 Intraperitoneal tumour dissemination assay

BALB/c nude mice purchased from Vital River Laboratories were raised in a specific pathogen‐free environment. Briefly, 1 × 10^7^ A2780 cells in which circRhoC was stably down‐regulated or control A2780 cells in 150 µL FBS‐free media were intraperitoneally injected into 5‐week‐old female mice to establish the model of intraperitoneal dissemination. Mice were killed 4 weeks after injection, and the tumour nodes and metastatic lesions were resected and measured. All animal experiments were carried out following the Guide for the Care and Use of Laboratory Animals published by the National Institute of Health and were approved by China Medical University Animal Care and Use Committee.

#### 2.6.1 Fluorescence in situ hybridization (FISH) assay

FISH was performed following the manufacturer's instructions (GenePharma). The adherent cells were seeded in a 24‐well plate at a density of 2.5 × 10^4^ cells/well (appropriately sized cover glass were pre‐loaded), and after incubating for 48 hours, cells were washed twice with PBS, fixed with 4% paraformaldehyde for 15 minutes at room temperature; then, 100 μL of 0.1% Buffer A was added into each well for 15 minutes at room temperature. After cells were washed twice with PBS, 100 μL of 2 × Buffer C was added into each well and incubated in a 37°C for 30 minutes. After aspirating 2 × Buffer C, cells were dehydrated for 3 minutes using an ethanol series (70%, 90% and 99%); then, absolute ethanol was aspirated and air‐dried, and 100 μL (2 μg) of pre‐denatured probe mixture was added per well, denatured at 73°C for 5 minutes and then incubated overnight at 37°C for 12‐16 hours. On the next day, the probe mixture was aspirated, 100 μL of 0.1% Buffer F was added into each well for 5 minutes. Then aspirate and add 100 μL 2 × Buffer C to each well for 5 minutes, again aspirate and add 100 μL 1 × Buffer C to each well for 5 minutes, and discard the washing solution. Add 100 μL of diluted DAPI and stain for 20 minutes in the dark; aspirate, wash, and observe under fluorescence microscope as soon as possible. The probe used for has‐circ‐0013519 was 5′‐ACAGAGCCAGCTCCACCACGTTGGA‐3′ and has‐miR‐302e: 5′‐AAGCATGGAAGCACTTA‐3′ (Hanbio Biotechnology).

### 2.7 RNA‐binding protein immunoprecipitation (RIP) assay

The RIP assay was performed using the Magna RIP RNA‐Binding Protein Immunoprecipitation Kit (Millipore) following the manufacturer's protocol. Briefly, A2780 cells at 80%‐90% confluency were lysed in RIP lysis buffer, and 100 µL of cell extract was incubated with RIP buffer containing magnetic beads conjugated to human anti‐VEGFA antibody or negative control normal rabbit IgG. The samples were incubated with proteinase K to digest proteins, and then, the immunoprecipitated RNA was isolated and subjected to qRT‐PCR analysis.

### 2.8 Dual‐luciferase reporter assay

HEK293T cells grew to 60% confluence. The wild‐type or mutated‐type PSI‐check2 dual‐luciferase vectors containing miR‐302e binding site on circRhoC (Hanbio Biotechnology) were cotransfected with miR‐302e mimics or scramble control into the HEK‐293T cells using Lipofectamine 2000 (Invitrogen). Cell extracts were prepared to measure the luciferase activity by the Dual‐Luciferase Reporter Assay System (Promega). The relative luciferase signal was represented by the normalization of firefly luciferase to that of renilla.

### 2.9 QRT‐PCR

Total cellular RNA was extracted using TRIzol (Vazyme Biotech Co. Ltd), dissolved in RNase‐free water, and cDNA was synthesized using the GoScript Reverse Transcription Kit (Promega). And then, qRT‐PCR was performed to amplify the target gene using GoTaq^®^ qPCR Master Mix (Promega) and relative expression of gene was normalized to 18S mRNA using the ΔΔCt method.

### 2.10 Western blotting

Ovarian cancer cells or tissues were lysed in radio‐immunoprecipitation assay buffer with protease inhibitors overnight, and the denatured protein samples were subjected to 10% or 12% sodium dodecyl sulphate‐polyacrylamide gel electrophoresis and electro‐transferred to PVDF membranes. Membranes were blocked in 3% bovine albumin serum (BSA) for 2 hours at room temperature and incubated with primary antibodies against β‐actin (1:5000) and VEGFA, TGF‐α and survivin (1:1000; Proteintech) overnight at 4°C, followed by anti‐rabbit secondary antibody (1:5000; Proteintech) for 2 hours, and then, protein bands were washed 3 times with PBS and then visualized using an enhanced chemiluminescence system (ECL, Santa Cruz Biotechnology).

### 2.11 Statistical analysis

Values are presented as the mean ± SD of three or more independent experiments or six mice per group. Two groups were compared using the Student's *t* test with SPSS 17.0 (SPSS). *P* < .05 was defined as statistical significance.

## 3 RESULTS

### 3.1 CircRhoC is overexpressed in ovarian cancer tissues

**Figure 1:**
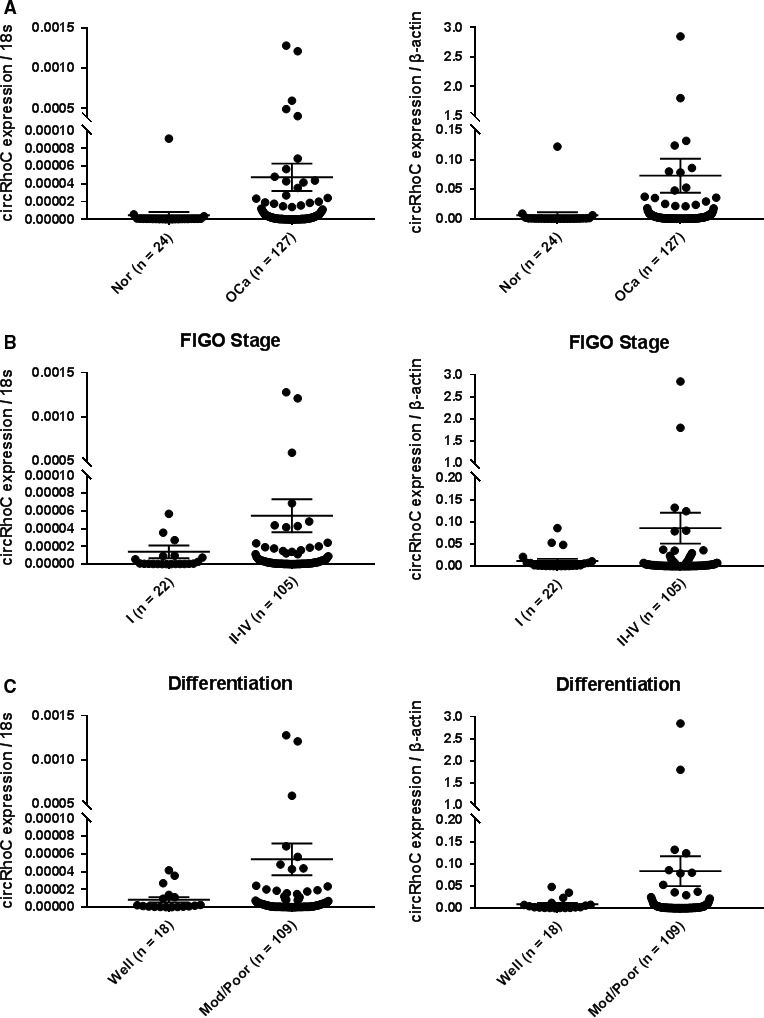
The prediction and expression of circRhoC in ovarian cancer tissues. Hsa_circ_0013549 (hereafter referred to as circRhoC) expression in the ovarian cancer tissues was significantly higher than that in the normal ovarian tissues (nor = normal ovarian tissues, OCa = ovarian cancer tissues; (A), *P* < .05) and positively associated with International Federation of Gynecology and Obstetrics (FIGO) stage (stage I vs. stage II‐IV; (B), *P* < .05), and it was lower in well‐differentiated group than in poor/moderate‐differentiated group (well vs. poor/moderate; (C), *P* < .05)

We used the bioinformatics website (www.circbase.org) and predicted the 14 possible circular RNA formed by RhoC mRNA and confirmed the circ_0013549 (hereafter referred to as circRhoC) expression in the ovarian cancer tissue as well as cell lines. Quantitative RT‐PCR was used to examine the expression of circRhoC in 127 ovarian cancer specimens and 24 normal ovarian tissues. CircRhoC was significantly overexpressed in the ovarian cancer tissues (Figure 1A, *P* < .05), and circRhoC expression was positively associated with International Federation of Gynecology and Obstetrics (FIGO) stage (stage I vs. stage II‐IV; Figure 1B, *P* < .05), and it was lower in well‐differentiated group than in poor/moderate‐differentiated group (well vs. poor/moderate; Figure 1C, *P* < .05). And details were provided in Tables S1 and S2.

### 3.2 CircRhoC increases cell viability in ovarian cancer cells

**Figure 2:**
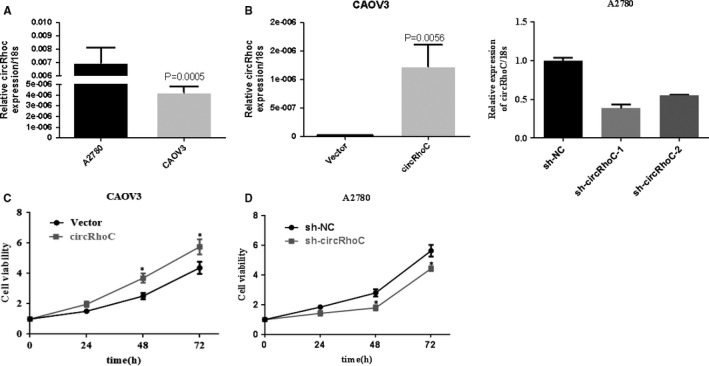
CircRhoC increases cell viability. The expression of circRhoC was highest in A2780 and lowest in CAOV3 relatively (A). The overexpressive plasmid transfected CAOV3 and increased the expression of circRhoC by 60 times compared with the control group. And transfection of shRNA decreased circRhoC expression in A2780, and the sh‐circRhoC‐1 had a higher silencing efficiency (B). Overexpression of circRhoC elevated the cell viability of CAOV3, and down‐regulated expression of cricRhoC showed in the opposite result in A2780 (C&D). Results are representative of three separate experiments; data are expressed as the mean ± standard deviation, *P < .05

In the panel of ovarian cancer cell lines tested, circRhoC was expressed at high levels in A2780 cells while relatively low levels in CAOV3 cells (Figure 2A). A plasmid overexpressing circRhoC was generated and transfected into CAOV3 cells, and the shRNA targeting the back‐spliced site with relative higher silencing efficiency was transfected into A2780 cells to destroy the circular structure and down‐regulate circRhoC (Figure 2B, *P < .05*). The MTT assay demonstrated that overexpressing circRhoC increased the viability of CAOV3 cells, while down‐regulation of circRhoC reduced the viability of A2780 cells (Figure 2C, *P* < .05).

### 3.3 CircRhoC enhances the migratory and invasive ability of ovarian cancer cells

**Figure 3:**
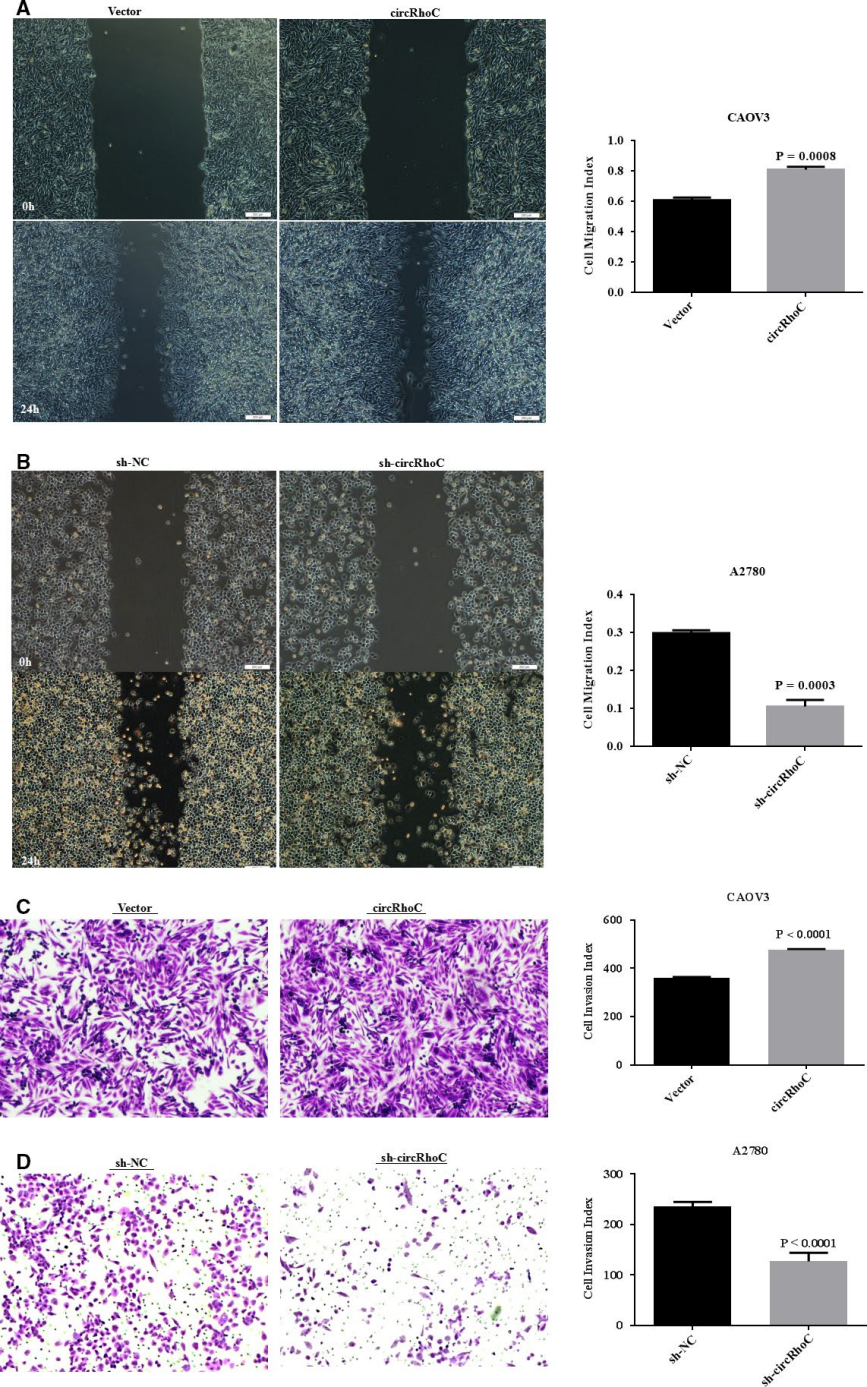
CircRhoC enhances the migratory and invasive ability. Up‐regulated expression of circRhoC increased the cell migration and invasion in CAOV3 (A&C) and sh‐circRhoC transfection reduced migratory and invasive ability in A2780 (B&D). Results are representative of three separate experiments; data are expressed as the mean ± standard deviation, *P < .05

Wound‐healing and Transwell migration assays demonstrated that overexpression of circRhoC promoted CAOV3 cell migration and invasion (Figure 3A,C, *P* < .05), whereas down‐regulation of circRhoC reduced the migratory and invasive ability of A2780 cells (Figure 3B,D, *P* < .05).

### 3.4 Down‐regulation of circRhoC attenuates intraperitoneal dissemination of ovarian cancer cells

**Figure 4:**
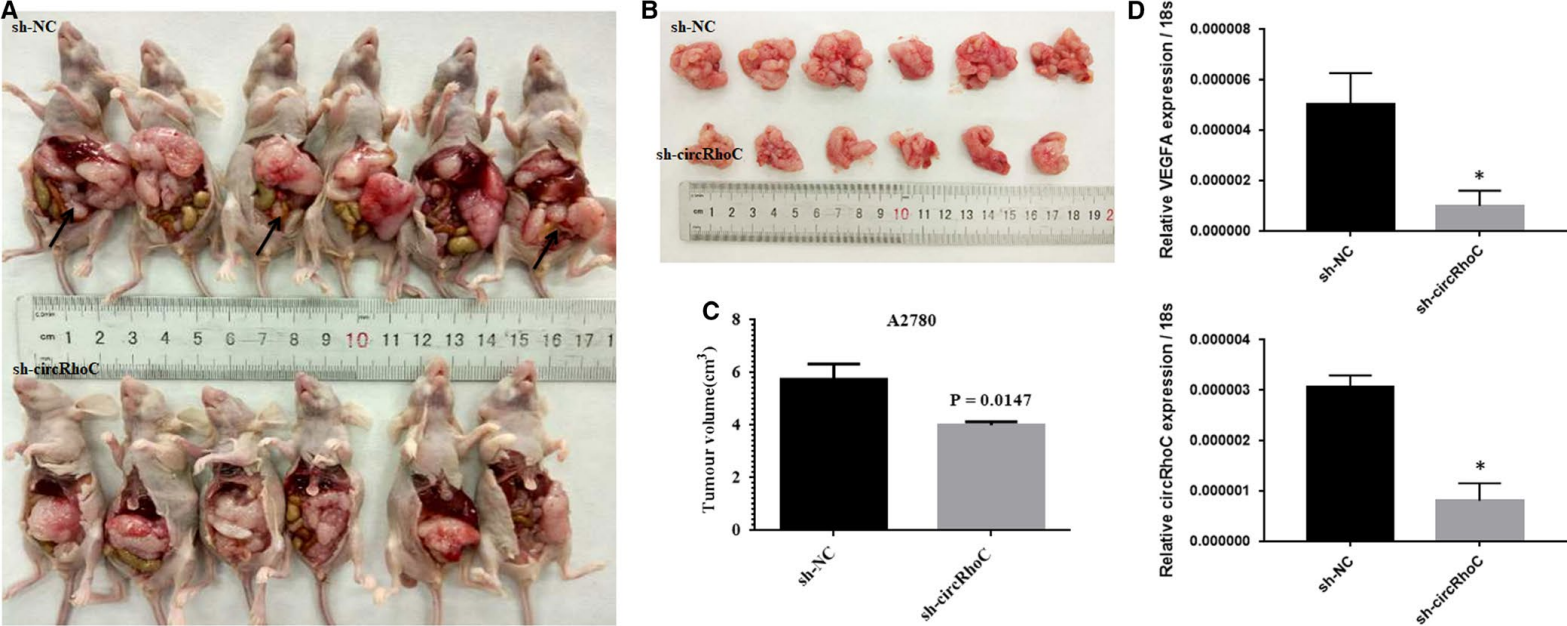
Down‐regulation of circRhoC inhibited intraperitoneal dissemination of ovarian cancer cells in vivo. The mice injected with A2780 transfected with sh‐circRhoC intraperitoneally showed smaller size and less range of metastatic lesions at macroscopic observation compared with the sh‐NC group (A‐C). Both circRhoC and VEGFA expression level were down‐regulated in sh‐circRhoC group than in sh‐NC group (D). Results are representative of three separate experiments; data are expressed as the mean ± standard deviation, *P < .05

Next, we investigated the effect of circRhoC in vivo using a mouse model of ovarian cancer. Mice were intraperitoneally injected with ovarian cancer cells transfected with the shRNA targeting circRhoC, which developed smaller tumour nodes and less numbers of tumour nodes in the mesentery compared to the control group injected with control A2780 cells. Moreover, the disseminated lesions were less spread throughout the mesentery in the mice injected with shRNA of circRhoC‐transfected cells (Figure 4A‐C). QRT‐PCR results showed that both circRhoC and VEGFA expression level was down‐regulated in sh‐circRhoC group (Figure 4D).

### 3.5 CircRhoC functions as a molecular sponge for miR‐302e to positively regulate VEGFA

**Figure 5:**
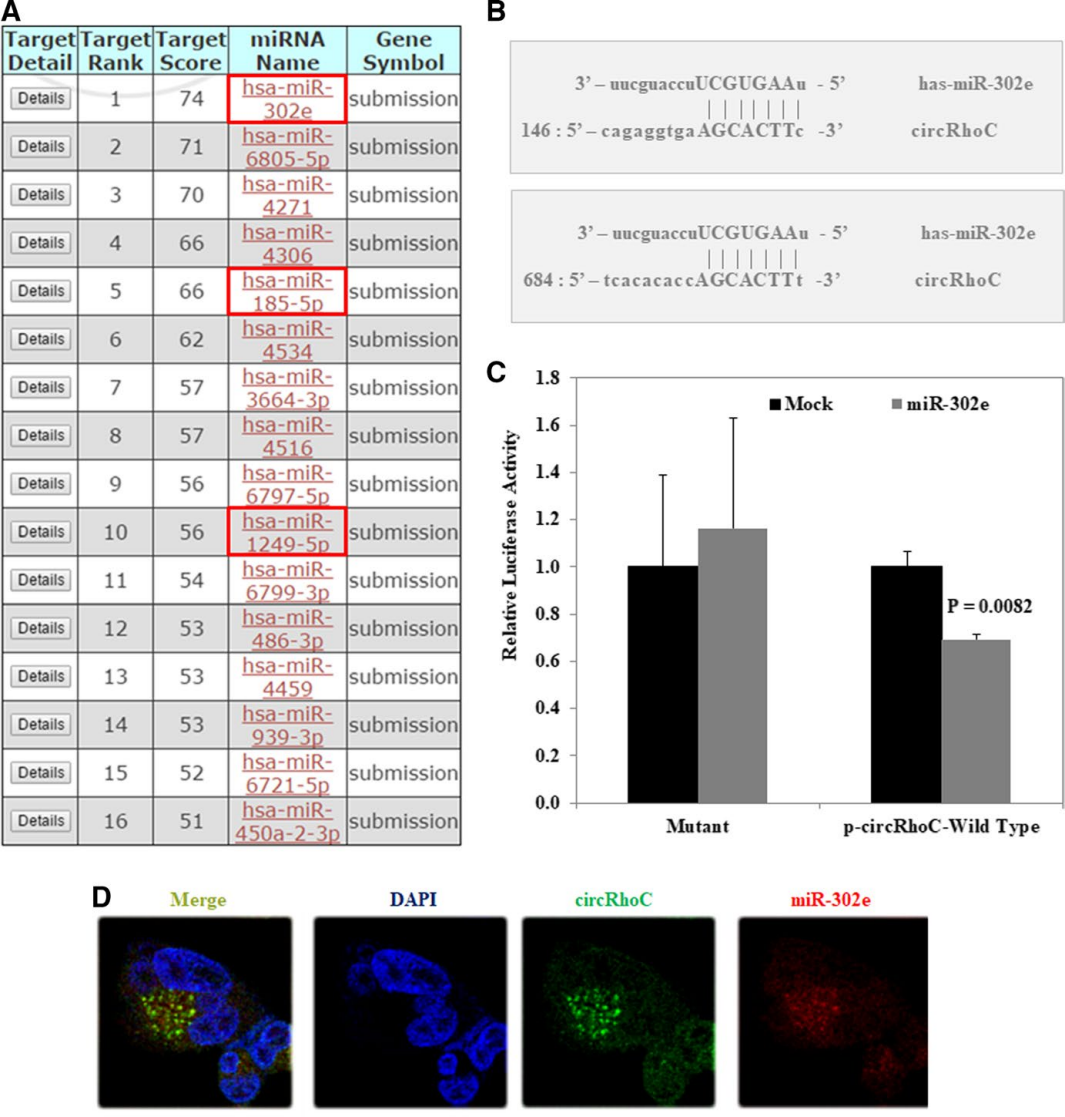
CircRhoC acted as a sponge of miR‐302e. The bioinformatics website predicted the potential binding sites of 16 miRNAs on circRhoC (A). The two binding sites for miR‐302e on circRhoC (B). Dual‐luciferase reporter assay indicated that miR‐302e binded directly to circRhoC (C). FISH assay showed that circRhoC co‐located with miR‐302e (D)

Bioinformatic analysis using the website (www.mirdb.org) to predict that circRhoC contains a binding site for 16 miRNAs including miR‐302e (Figure 5A). And there are two binding sites for miR‐302e on circRhoC (Figure 5B). Using a dual‐luciferase reporter assay, we confirmed that miR‐302e binds directly to circRhoC (Figure 5C, *P* < .05). In order to further research whether circRhoC co‐located with miR‐302e, we performed FISH assay, and we found circRhoC was co‐located with miR‐302e (Figure 5D).

**Figure 6:**
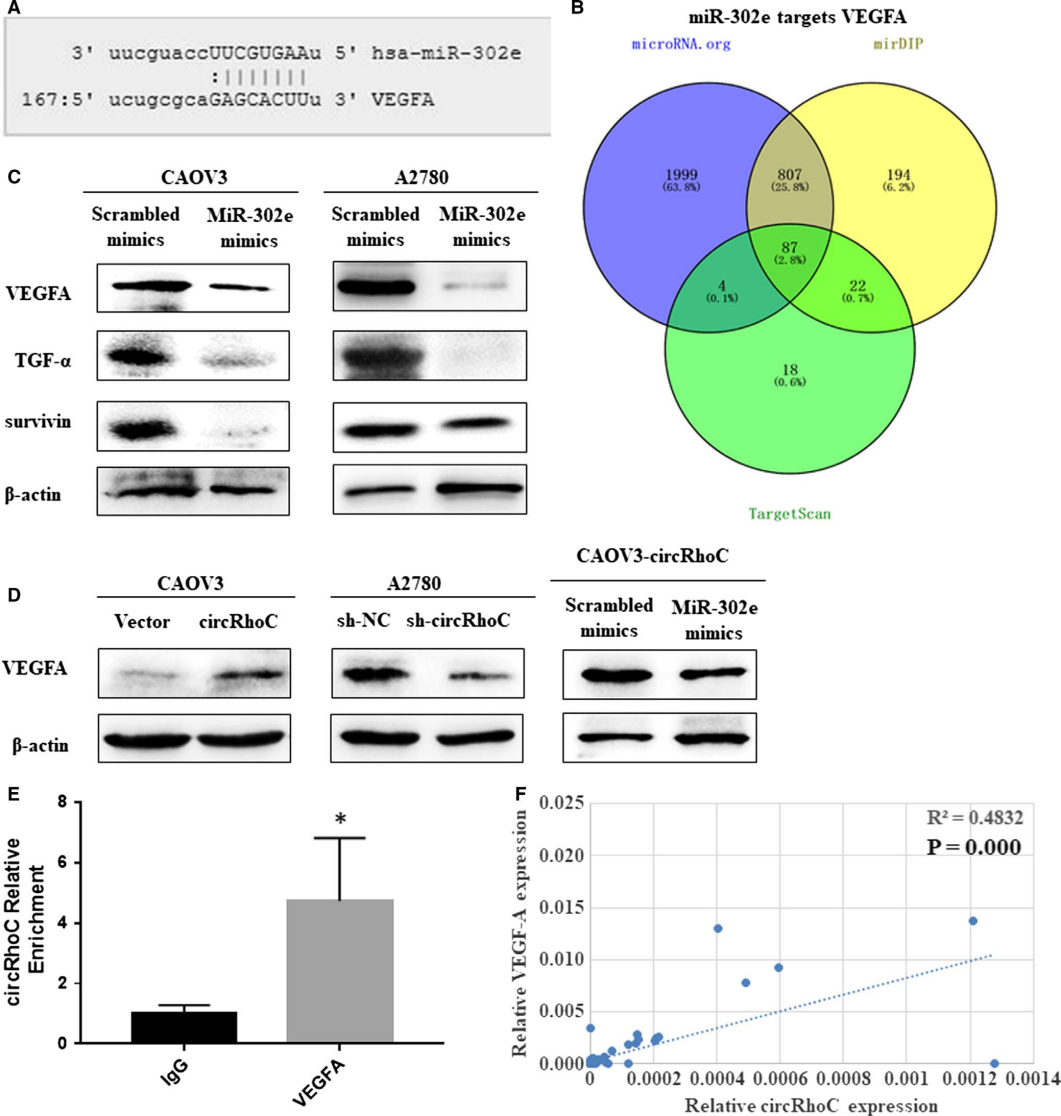
CircRhoC sponged miR‐302e, released VEGFA and elevated other proteins. Bioinformatic analyses (mirDIP, TargetScan, and microRNA.org) predicted that miR‐302e shares 87 binding sites (B), which contains VEGFA (A). Transfecting with miR‐302e down‐regulated VEGFA, TGF‐α and survivin in CAOV3 and A2780 cells (C). Overexpression of circRhoC increased VEGFA protein expression in CAOV3. And A2780 transfected with sh‐circRhoC resulted in lower VEGFA expression compared with the sh‐NC group (D). And overexpression of miR‐302e reversed the up‐regulation of VEGFA by circRhoC (D). circRhoC could directly bind with VEGFA protein (E). VEGFA expression level in ovarian cancer tissues was positively associated with circRhoC expression (*P* = .000, F)

Bioinformatic analyses (mirDIP, TargetScan, and microRNA.org) predicted that miR‐302e shares 87 binding sites, which contains VEGFA mRNA 3′ UTR (Figure 6A,B). Western blotting confirmed that ovarian cancer cells transfected with a miR‐302e mimic expressed lower levels of VEGFA, TGF‐α and survivin protein (Figure 6C). Moreover, overexpression of circRhoC up‐regulated VEGFA protein expression, whereas down‐regulating circRhoC had the opposite effect (Figure 6D). In addition, and transfection of miR‐302e into circRhoC‐overexpressing CAOV3 cells reversed circRhoC‐induced up‐regulation of VEGFA (Figure 6D). RIP assays were performed to assess if VEGFA protein interacts with RNA. RNA obtained from the RIP assay using a VEGFA antibody was subjected to qPCR analysis, which demonstrated enrichment of the circRhoC (Figure 6E). Besides, VEGFA expression level in ovarian cancer tissues was positively associated with circRhoC expression (*P* = .000, Figure 6F).

## 4 DISCUSSION

The purpose of this study was to explore the role and mechanism of action of circRhoC, a putative circRNA formed from *RhoC*, in ovarian cancer. Using a large number of patient samples, we confirmed circRhoC was expressed at high levels in ovarian cancer tissues compared to normal ovarian tissues. Furthermore, we up‐regulated circRhoC using an expression vector in CAOV3 cells, and two kinds of shRNAs targeting the back‐spliced sites were designed, and the shRNA with high silencing efficiency selected by qRT‐PCR was used to destroy the circRhoC structure in A2780 cells by stable transfection. Overexpression of circRhoC increased cell viability, migration and invasion, whereas destroying the structure of circRhoC had the opposite effects in vitro and reduced intraperitoneal dissemination of ovarian cancer cells in vivo. Therefore, these results demonstrate that circRhoC, a circular RNA formed from *RhoC*, is overexpressed and promotes tumour progression in ovarian cancer.

CircRNAs are inherently resistant to exonuclease‐mediated decay and can act as miRNA sponges to prevent miRNAs destabilizing or inhibiting translation of their target mRNAs. Therefore, circRNAs may exert a stronger regulatory role on miRNAs than mRNAs or lncRNAs. Altered expression of a variety of miRNAs has been shown to play important roles in the development of cancer, and the regulatory network formed by circRNAs and miRNAs has become a hotspot of research. For example, Thomas et al demonstrated ciRS‐7 is expressed in the mouse brain and strongly inhibits miR‐7.^24^ Moreover, circ‐ABCB10 promotes progression in breast cancer by acting as a miR‐1271 sponge.^25^

Our bioinformatic analysis revealed circRhoC contains putative binding sites for multiple miRNAs, among which mir‐185‐5p, mir‐302e and mir‐1249‐5p may also bind VEGFA's 3′UTR simultaneously. Further in HEK293 cells, three miRNAs were cotransfected with either circRhoC or the corresponding mutants, respectively. Using luciferase reporter assay, we confirmed that circRhoC acts as a miR‐302e sponge, and through FISH assay, we also found that circRhoC was co‐located with miR‐302e. Several studies have shown miR‐302 inhibits proliferation and acts as a tumour suppressor in a variety of cancers,^26^, ^27^, ^28^ including ovarian cancer.^29^ MiR‐302e was transfected into ovarian cancer cells CAOV3 and A2780, and the expression of VEGFA, TGF‐α and survivin was down‐regulated compared with the control group, suggesting the inhibitory effect of miR‐302e on the occurrence and development of ovarian cancer. In addition, we found that circRhoC overexpression promotes VEGFA expression in ovarian cancer cells, and VEGFA expression level in ovarian cancer tissues was positively associated with circRhoC expression; moreover, through RIP assay, we suggest that circRhoC could directly bind with VEGFA protein. We further prove that the overexpression of miR‐302e could reverse up‐regulation of VEGFA in cells stably overexpressing circRhoC. Therefore, we conclude circRhoC acts as miR‐302e sponge and positively regulate *VEGFA*.

VEGFA is a well‐characterized growth and survival factor for vascular endothelial cells that is overexpressed in a wide range of tumours, including ovarian cancer.^30^ VEGFA can enhance cell invasion by inducing angiogenesis and activating matrix metalloproteinases^31^ and increase cell viability.^32^ Moreover, VEGFA can also protect against apoptosis in renal clear cell carcinoma.^33^ A number of anti‐VEGF therapies exist and have been proposed for ovarian cancer.^34^ Therefore, study of the mechanisms that regulate VEGFA is important. And the oncogenetic role of TGF‐α and survivin in ovarian cancer cells has also been confirmed.^35^, ^36^, ^37^

## 5 CONCLUSION

RhoC functions as an oncogene in ovarian cancer. We describe a novel molecular mechanism by which RhoC forms a circRNA that not only sponges miR‐302e to positively regulate VEGFA, but may also directly bind and modulate VEGFA expression, which may promote tumorigenicity and progression in ovarian cancer. The circRhoC/miR‐302e/VEGFA axis may provide a novel therapeutic target for ovarian cancer.

## CONFLICT OF INTEREST

The authors have no conflicts of interest to declare.

## AUTHOR CONTRIBUTIONS

Yang Zhao and Li‐Li Wang conceived the study, analysed interpretation and wrote the manuscript. Li‐Li Wang, Zhi‐Hong Zong, Yao Liu, Xue Guan and Shuo Chen performed the experiments and analysed the data. All authors read and approved the final manuscript.

## ETHICAL APPROVAL

The research protocol was approved by the China Medical University Ethics Committee (No. 2014‐27).

## Data Availability

The data sets used and/or analysed during the current study are available from the corresponding author on reasonable request.
