# Supplementary material for: CircRhoC promotes tumorigenicity and progression in ovarian cancer by functioning as a miR‐302e sponge to positively regulate VEGFA
Source: J Cell Mol Med. 2019 Oct 22;23(12):8472–81. doi: 10.1111/jcmm.14736 (PMC6850961; doi:10.1111/jcmm.14736)
Supplement: Supplementary file 1 [file JCMM-23-8472-s001.doc]

**Supplementary Table 1:** circRhoC expression in normal ovary and ovarian carcinoma tissues

| **Groups** | **N** | **circRhoC expression / 18s** | ***P* value** |
| --- | --- | --- | --- |
|
| Normal ovary | 24 | 4.61E-06 ± 1.85E-05 | ***0.004*** |
| Ovarian carcinoma | 127 | 4.76E-05 ± 0.000174 |  |

| **Groups** | **N** | **circRhoC expression / β-actin** | ***P* value** |
| --- | --- | --- | --- |
|
| Normal ovary | 24 | 0.006109 ± 0.024863 | ***0.012*** |
| Ovarian carcinoma | 127 | 0.072981 ± 0.356440 |  |

Bold and Italics means P < 0.05.

**Supplementary Table 2:** Correlation of circRhoC expression with different clinicopathological features of ovarian carcinoma

| **Clinicopathological features** | **N** | **circRhoC expression / 18s** | | ***P* value** |
| --- | --- | --- | --- | --- |
|
| **The pathology types** |  |  | | 0.169 |
| Serous carcinoma | 100 | 5.22E-05 ± 0.000194 | |  |
| The other pathology types | 27 | 3.07E-05 ± 5.69E-05 | |  |
| **Age** |  |  | | 0.397 |
| ≤ 52 | 66 | 4.37E-05 ± 0.000168 | |  |
| > 52 | 61 | 5.18E-05 ± 0.000182 | |  |
| **FIGO stages** |  |  | | ***0.021*** |
| I | 22 | 1.39E-05 ± 3.32E-05 | |  |
| II-IV | 105 | 5.47E-05 ± 0.000190 | |  |
| **Differentiation classification** |  |  | | ***0.007*** |
| Well | 18 | 8.39E-06 ± 1.31E-05 | |  |
| Mod + Poor | 109 | 5.41E-05 ± 0.000187 | |  |
| **CA125 expression** |  |  | |  |
| ≤ 35 U/ml | 9 | 4.37E-05 ± 8.01E-05 | | 0.448 |
| > 35 U/ml | 118 | 4.79E-05 ± 0.000180 | |  |
| Bold and Italics means P < 0.05. | | | | |
| **Clinicopathological features** | **N** | **circRhoC expression /β-actin** | | ***P* value** |
|
| **The pathology types** |  | |  | 0.449 |
| Serous carcinoma | 100 | | 0.071572 ± 0.353516 |  |
| The other pathology types | 27 | | 0.078197 ± 0.193662 |  |
| **Age** |  | |  | 0.376 |
| ≤ 52 | 66 | | 0.081686 ± 0.376521 |  |
| > 52 | 61 | | 0.063562 ± 0.262141 |  |
| **FIGO stages** |  | |  | ***0.019*** |
| I | 22 | | 0.011684 ± 0.022120 |  |
| II-IV | 105 | | 0.085824 ± 0.356777 |  |
| **Differentiation classification** |  | |  | ***0.014*** |
| Well | 18 | | 0.008650 ± 0.013467 |  |
| Mod + Poor | 109 | | 0.083604 ± 0.350377 |  |
| **CA125 expression** |  | |  |  |
| ≤ 35 U/ml | 9 | | 0.079493 ± 0.189509 | 0.461 |
| > 35 U/ml | 118 | | 0.072484 ± 0.334111 |  |
| Bold and Italics means P < 0.05. | | | | |

**Supplementary Table 3:** CircRhoC overexpression vector sequence

TGTTTTGACCTCCATAGAAGACACCGACTCTACTAGAGGATCTATTTCCGGTGAATTCAAAGTGCTGAGATTACAGGCGTGAGCCACCACCCCCGGCCCACTTTTTGTAAAGGTACGTACTAATGACTTTTTTTTTATACTTCAGCTCGAGGTGGAGCTGGCTCTGTGGGACACAGCAGGGCAGGAAGACTATGATCGACTGCGGCCTCTCTCCTACCCGGACACTGATGTCATCCTCATGTGCTTCTCCATCGACAGCCCTGACAGCCTGGAAAACATTCCTGAGAAGTGGACCCCAGAGGTGAAGCACTTCTGCCCCAACGTGCCCATCATCCTGGTGGGGAATAAGAAGGACCTGAGGCAAGACGAGCACACCAGGAGAGAGCTGGCCAAGATGAAGCAGGAGCCCGTTCGGTCTGAGGAAGGCCGGGACATGGCGAACCGGATCAGTGCCTTTGGCTACCTTGAGTGCTCAGCCAAGACCAAGGAGGGAGTGCGGGAGGTGTTTGAGATGGCCACTCGGGCTGGCCTCCAGGTCCGCAAGAACAAGCGTCGGAGGGGCTGTCCCATTCTCTGAGATCCCCAAGGCCTTTCCTACATGCCCCCTCCCTTCACAGGGGTACAGAAATTATCCCCCTACAACCCCAGCCTCCTGAGGGCTCCATGCTGAAGGCTCCCATTTTCAGTTCCCTCCTGCCCAGGACTGCATTGTTTTCTAGCCCCGAGGTGGTGGCACGGGCCCTCCCTCCCAGCGCTCTGGGAGCCACGCCTATGCCCTGCCCTTCCTCAGGGCCCCTGGGGATCTTGCCCCCTTTGACCTTCCCCAAAGGATGGTCACACACCAGCACTTTATACACTTCTGGCTCACAGGAAAGTGTCTGCAGTAGGGGACCCAGAGTCCCAGGCCCCTGGAGTTGTTTTCGGCAGGGGCCTTGTCTCTCACTGCATTTGGTCAGGGGGGCATGAATAAAGGCTACAGGCTCCAACGTGCTCGAGGTAAGAAGCAAGGAAAAGAATTAGGCTCGGCACGGTAGCTCACACCTGTAATCCCAGCAGGATCCATCGATACTAGTAAGGATCTGCGATCGCTCCGGTGCCCG

The yellow region were the sequences of circRhoC.
